# Camelid VHHs as a Versatile Platform for Combating Viral Infections

**DOI:** 10.3390/ph19091408

**Published:** 2026-09-06

**Authors:** Mohammed Mufrrih, Thamir A. Alandijany

**Affiliations:** 1 Department of Medical Laboratory Sciences, Faculty of Applied Medical Sciences, King Abdulaziz University, P.O. Box 80324, Jeddah 21589, Saudi Arabia; mmufrrih@kau.edu.sa; 2 Special Infectious Agents Unit, King Fahd Medical Research Center, King Abdulaziz University, P.O. Box 128442, Jeddah 21362, Saudi Arabia

**Keywords:** camelid VHH nanobodies, single-domain antibodies, respiratory viruses, influenza, coronaviruses, RSV

## Abstract

Viral infections continue to pose a major global health challenge because of rapid viral evolution, immune escape, and the emergence of novel pathogens. This review examines the biological characteristics, antiviral mechanisms, production strategies, and therapeutic potential of camelid VHH as next-generation antiviral agents. The review synthesizes current evidence on the structural and functional properties of VHH, their methods of generation and engineering, and their applications against a broad range of respiratory and non-respiratory viruses. Camelid VHH nanobodies exhibit high specificity and affinity, exceptional physicochemical stability, efficient tissue penetration, and the unique ability to recognize conserved and cryptic epitopes that are often inaccessible to conventional antibodies. Engineering approaches, including multivalent, bispecific, Fc-fused, and half-life-extended constructs, further enhance their antiviral potency and pharmacokinetic properties. Evidence from preclinical studies demonstrates potent antiviral activity against multiple viral pathogens through mechanisms that include blocking viral attachment and fusion, inhibiting intracellular replication, targeting conserved viral proteins, and reducing the likelihood of viral escape. The review also highlights emerging advances in recombinant production platforms, artificial intelligence-assisted nanobody design, targeted delivery technologies, and One Health applications. Collectively, the available evidence indicates that camelid VHH represent a highly versatile and adaptable platform for antiviral therapeutics and diagnostics, with considerable potential to support pandemic preparedness and the development of broad-spectrum interventions against current and emerging viral diseases.

## 1. Introduction

The continuous emergence and re-emergence of viral infections, driven by rapid viral evolution and immune escape mechanisms, necessitates the development of innovative antiviral strategies. Although conventional antibody-based therapies have demonstrated significant clinical value, their large molecular size, limited tissue penetration, production complexity, and restricted access to cryptic viral epitopes remain important limitations. These challenges have intensified interest in alternative antibody formats with enhanced structural and functional versatility.

Camelid-derived variable domains of heavy-chain-only antibodies (VHHs) and the isolated VHH (commonly termed nanobodies) have emerged as promising next-generation antiviral therapeutics [1,2,3]. Despite their small size, they maintain high specificity and affinity comparable to conventional antibodies while offering unique advantages [4,5]. Their elongated complementarity-determining region 3 (CDR3) enables access to hidden or conserved viral epitopes that are often inaccessible to conventional antibodies, allowing efficient targeting of viral glycoproteins, receptor-binding domains, and fusion-associated structures [4,5,6,7]. In addition, VHHs exhibit remarkable physicochemical stability, high solubility, and compatibility with cost-effective microbial expression systems such as *Escherichia coli (E. Coli)* and yeast [8,9]. Their small size enhances tissue penetration and supports alternative delivery routes, including inhalation and intranasal administration, making them particularly attractive for respiratory viral infections [8,9]. Moreover, They can be engineered into multivalent, multispecific, or Fc-fused constructs to improve neutralization potency, broaden antiviral coverage, and enhance pharmacokinetics [5,10]. These engineered formats have demonstrated strong potential against several viral pathogens, including SARS-CoV-2, influenza virus, respiratory syncytial virus, HIV, and others [7,11,12]. Collectively, these properties position camelid VHH as a versatile and rapidly adaptable platform for antiviral therapeutics and diagnostics. This review explores the structural features, antiviral mechanisms, and therapeutic potential of camelid VHH against viral infections.

## 2. Biology and Structural Features of Camelid VHHs

### 2.1. Origin and Immunological Basis of Heavy-Chain Antibodies

Heavy-chain-only antibodies (HCAbs) were first identified in camelids as a naturally occurring immunoglobulin isotype that lacks light chains [1,2]. Structurally, these antibodies are composed solely of heavy chains and are devoid of the CH1 domain, which in conventional immunoglobulins mediates interaction with light chains [4,5]. As a result, the antigen-binding site of HCAbs is formed exclusively by a single variable domain, known as VHH [1,3,5].

From an immunological perspective, HCAbs are fully functional and participate in antigen recognition and immune defense in parallel with conventional IgG antibodies [13,14]. The VHH domain has evolved specific amino acid substitutions within its framework regions to compensate for the absence of light chains, ensuring proper folding, solubility, and antigen-binding capability [15,16,17]. These adaptations allow VHH to maintain high affinity while functioning independently as monomeric units [15,16,17]. Importantly, their simplified genetic and structural organization facilitates rapid cloning, selection, and recombinant expression, which has significantly accelerated their development for biomedical applications [5,18].

### 2.2. Comparison with Conventional Antibody Formats

**Figure 1:**
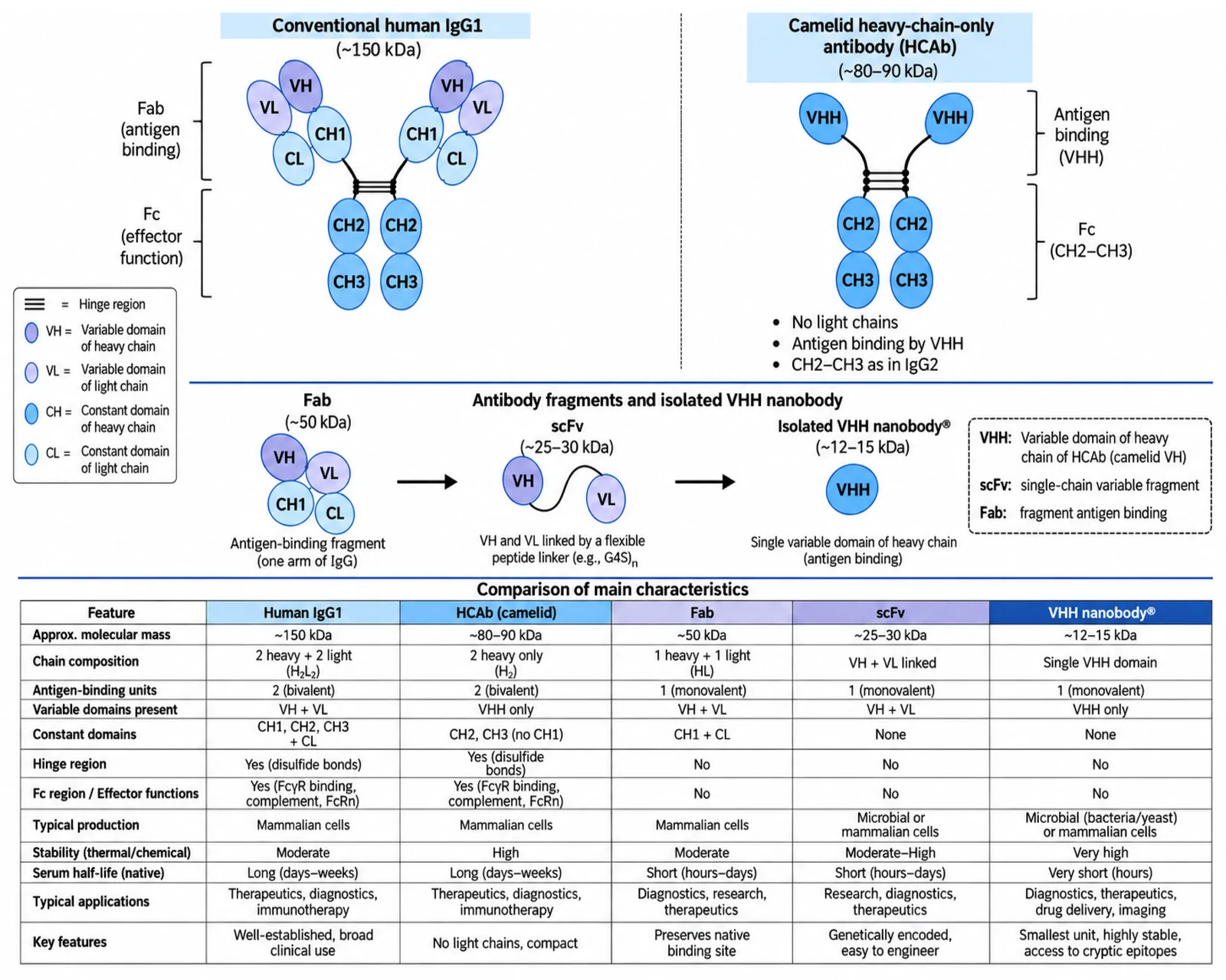
Schematic structures and comparison of conventional human IgG1, camelid heavy chain only antibody (HCAb), antibody fragments, and VHH. Conventional human IgG1 consists of two heavy and two light chains, with paired VH and VL domains forming the antigen binding regions and CH2–CH3 domains forming the Fc region. In contrast, camelid HCAbs lack light chains and the CH1 domain, with antigen recognition mediated exclusively by the VHH domain. Representative antibody fragments are shown below, including the Fab fragment, comprising VH, VL, CH1, and CL domains; the single chain variable fragment (scFv), in which VH and VL domains are connected by a flexible peptide linker; and the VHH, representing the single antigen binding variable domain of an HCAb. The table summarizes the principal structural and functional characteristics of human IgG1, camelid HCAb, Fab, scFv, and VHH, including molecular mass, chain composition, antigen binding valency, domain organization, Fc associated effector functions, production, stability, serum half life, typical applications, and key distinguishing features.

Camelid VHHs differ substantially from conventional IgG antibodies in terms of size, structural complexity, and functional behavior (Figure 1). While IgG molecules are large (~150 kDa) heterotetrameric proteins composed of two heavy and two light chains, VHHs are compact (~12–15 kDa), single-domain entities [6]. VHHs originate from the variable domains of HCAbs, predominantly represented by the IgG2 and IgG3 subclasses, which lack light chains and CH1 domains but retain an Fc region. Once isolated from the HCAb, the VHH constitutes an independently folding single antigen-binding domain of approximately 12–15 kDa and lacks the Fc region and its associated effector functions [3,5,7,18,19].

The small size of VHH can facilitate diffusion and access to sterically constrained environments and is particularly advantageous for local delivery. However, this property is format and context dependent and should not be regarded as an inherent advantage over other antibody fragments. Fab and scFv formats are also substantially smaller than intact IgG and offer their own balances of tissue distribution, target accessibility, and pharmacokinetic behavior [20,21,22].

VHH possess high intrinsic stability and solubility can support retention of functional activity under challenging physicochemical conditions under conditions that would typically denature conventional antibodies [23]. Third, their monomeric nature facilitates efficient recombinant production in microbial systems reducing manufacturing complexity and cost, and provides considerable flexibility for the generation of multivalent, multispecific, or fusion constructs [24,25,26].

In terms of antigen binding, VHH exhibit comparable affinity to conventional antibodies but differ in their mode of interaction [27,28,29]. Whereas IgG antibodies primarily recognize flat or exposed epitopes, VHH excel at binding concave, hidden, or enzymatic surfaces due to their elongated CDR3 loops and flexible paratope architecture [27,28,29]. This structural property can expand the accessible epitope repertoire for particular microbial targets, but does not confer a universal binding or neutralization advantage over Fab, scFv, or VH formats. Rather, the optimal format depends on the topology and accessibility of the target epitope, the required mechanism of neutralization, and whether avidity or multivalent engagement is advantageous.

However, their small size also results in rapid renal clearance, which may limit their circulatory half-life in vivo [30,31]. This limitation can be addressed through bioengineering strategies such as Fc fusion, PEGylation, or albumin binding, enabling optimization of pharmacokinetic properties without compromising their unique binding capabilities [32,33,34,35,36].

## 3. Functional Advantages of VHHs in Infectious Diseases

The unique structural and biochemical properties of VHHs translate into a set of functional advantages that are particularly valuable in the context of infectious diseases. Their ability to access inaccessible antigenic sites, maintain activity under challenging conditions, penetrate tissues efficiently, and undergo extensive molecular engineering positions VHHs as a highly adaptable platform for both therapeutic and diagnostic applications. These features are especially relevant when targeting pathogens characterized by high mutation rates, complex surface architectures, or intracellular life cycles.

### 3.1. Recognition of Cryptic and Conserved Epitopes

A defining functional advantage of VHHs is their ability to recognize cryptic, recessed, or conformational epitopes that are often inaccessible to conventional antibodies [29,37,38,39,40]. This capability is primarily driven by their elongated complementarity-determining region 3 (CDR3), which can extend into narrow grooves, enzyme active sites, or shielded regions within antigenic structures [29,37,38,39,40]. As a result, VHHs can bind to functionally critical regions of microbial proteins, including viral receptor-binding domains, fusion machinery, and bacterial toxin active sites [29,37,38,39,40].

Importantly, many of these cryptic epitopes are highly conserved due to structural or functional constraints, making them less prone to antigenic variation [39,41,42,43]. Targeting such conserved regions enhances the likelihood of broad-spectrum activity and reduces the risk of immune escape. This is particularly advantageous in viral infections, where antigenic drift and shift frequently compromise the efficacy of conventional antibodies [39,41,42,43]. By engaging conserved epitopes, VHH can achieve cross-strain or even cross-species reactivity, thereby expanding their therapeutic utility [39,41,42,43].

### 3.2. Stability Under Harsh Conditions

Camelid VHHs exhibit exceptional physicochemical stability, retaining their structural integrity and binding activity under conditions that typically denature conventional antibodies [44,45,46,47]. They are resistant to high temperatures, extreme pH levels, and the presence of denaturing agents, owing to their compact structure and robust intramolecular interactions [44,45,46,47].

This intrinsic stability enables VHHs to be utilized in diverse and challenging environments, including resource-limited settings where cold-chain logistics are not feasible [4]. Furthermore, their resilience to processes such as lyophilization and aerosolization makes them particularly suitable for non-invasive delivery formats, including inhalable powders and nasal sprays [19,48]. Such properties are highly advantageous for infectious disease applications, where rapid deployment, environmental robustness, and ease of administration are critical.

### 3.3. Tissue Penetration and Delivery Flexibility

The small molecular size of VHH (~12–15 kDa) significantly enhances their ability to penetrate tissues and access anatomically restricted sites [5,21,49]. Unlike conventional IgG antibodies, which may be limited by their size and steric constraints, VHHs can diffuse more efficiently into dense tissues, mucosal surfaces, and even intracellular compartments when appropriately engineered [5,21,49].

This improved tissue distribution is particularly relevant for infections localized to mucosal barriers, such as respiratory, gastrointestinal, and urogenital pathogens [6,7,12,50]. VHHs can be administered via alternative routes, including intranasal, inhalational, or oral delivery, enabling targeted intervention at the primary site of infection. Such localized delivery not only enhances therapeutic efficacy but also minimizes systemic exposure and potential side effects pathogens [6,7,12,50]. Additionally, their rapid systemic clearance, while a limitation in some contexts, can be advantageous in diagnostic imaging or short-acting therapeutic applications where quick elimination is desirable [51,52,53].

### 3.4. Engineering Potential

One of the most powerful aspects of VHH lies in their amenability to molecular engineering. Their simple structure and genetic tractability allow for the design of customized constructs with enhanced functionality and optimized pharmacological properties.

#### 3.4.1. Multivalent and Bispecific Constructs

VHHs can be linked together to form multivalent or multispecific constructs, thereby increasing binding avidity and enabling simultaneous targeting of multiple epitopes or antigens [8,54,55]. Multivalent VHHs enhance neutralization potency through avidity effects, while bispecific or biparatopic designs can engage distinct antigenic sites, reducing the likelihood of pathogen escape and improving therapeutic breadth [38,56,57]. These strategies are particularly valuable against highly mutable viruses and complex microbial targets [38,56,57].

#### 3.4.2. Fc-Fusion and Half-Life Extension

To overcome rapid renal clearance, VHHs can be fused to Fc domains of immunoglobulins or engineered to bind serum albumin [34,56,58]. Fc-fusion not only extends circulatory half-life but also introduces effector functions such as antibody-dependent cellular cytotoxicity (ADCC) and complement activation, thereby enhancing antimicrobial activity [59,60,61]. Alternative strategies, including PEGylation or albumin-binding domains, further improve pharmacokinetics while maintaining the inherent advantages of the VHH scaffold [32,33,35,36]. Such modifications, however, increase molecular size and may alter tissue distribution, valency, and manufacturability. Consequently, selection Fc-containing formats should be guided by the intended target, route of administration, required duration of exposure, and desired mechanism of action.

Collectively, these functional attributes, ranging from deep epitope recognition to engineering versatility, underscore the potential of camelid VHHs as a next-generation platform for combating infectious diseases. Their adaptability allows them to be tailored to diverse pathogens and clinical needs, positioning them at the forefront of innovative antimicrobial strategies.

## 4. Production and Engineering of Camelid VHHs

**Figure 2:**
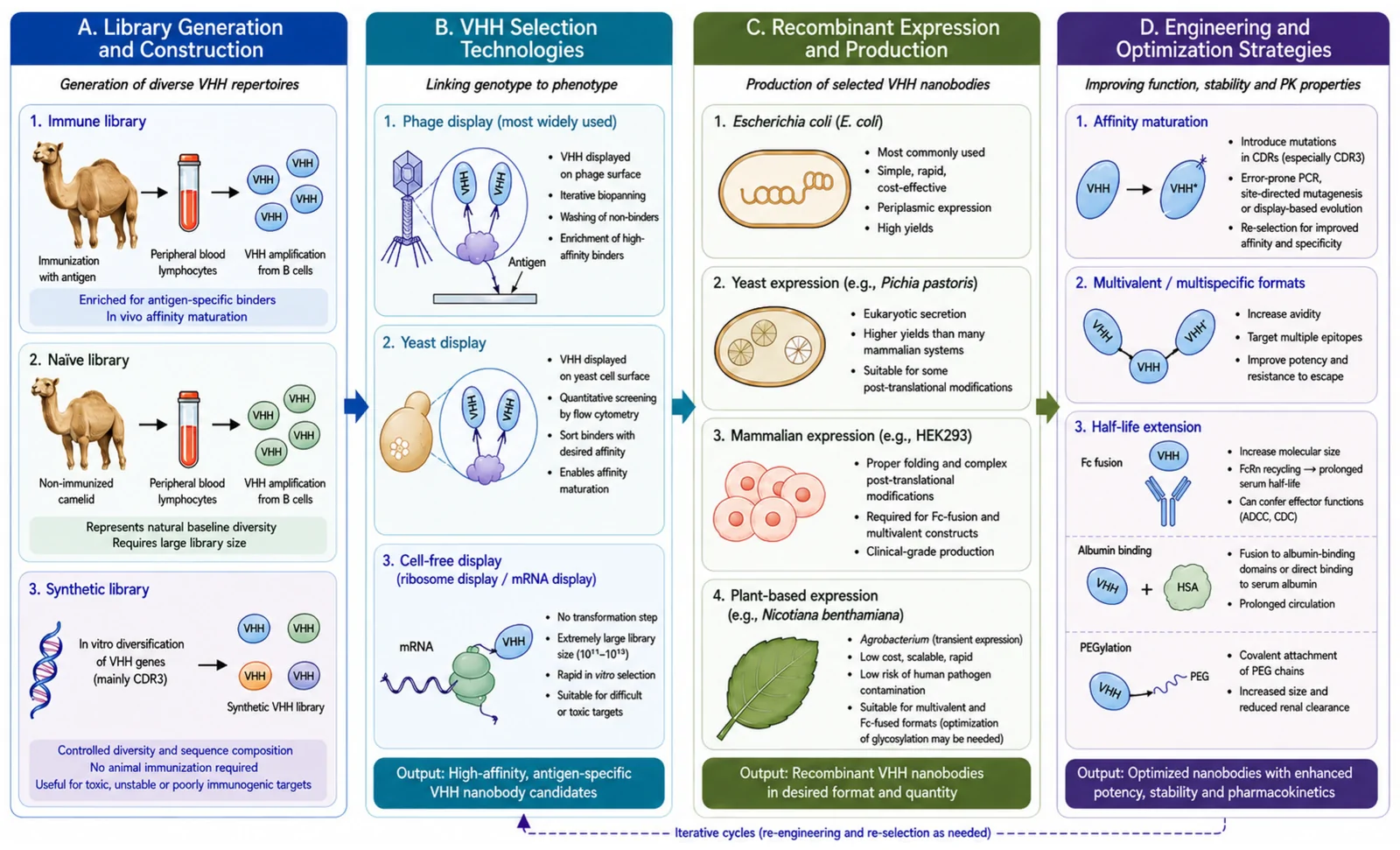
Workflow for generation, selection, recombinant production, and engineering of camelid VHH. The VHH development pipeline comprises four interconnected stages. (**A**) Library generation and construction: VHH repertoires can be derived from immune libraries generated following camelid immunization, naïve libraries obtained from nonimmunized camelids, or synthetic libraries generated through controlled in vitro diversification of VHH sequences. (**B**) VHH selection technologies: antigen specific VHH candidates are enriched using genotype to phenotype selection platforms, including phage display, yeast display, and cell free approaches such as ribosome or mRNA display. (**C**) Recombinant expression and production: selected VHH can be produced using bacterial, yeast, mammalian, or plant based expression systems, with platform selection depending on the required yield, protein complexity, post translational modifications, and intended application. (**D**) Engineering and optimization: selected VHHs can undergo affinity maturation, conversion into multivalent or multispecific formats, and half life extension through strategies such as Fc fusion, albumin binding, or PEGylation. Optimized candidates may subsequently undergo iterative re engineering and re selection to further improve affinity, specificity, potency, stability, and pharmacokinetic properties. Abbreviations: VHH, variable domain of a camelid heavy chain only antibody; CDR, complementarity determining region; Fc, fragment crystallizable; HSA, human serum albumin; PEG, polyethylene glycol; PK, pharmacokinetics.

The successful translation of camelid VHHs into therapeutic and diagnostic applications relies on efficient strategies for their generation, selection, and optimization (Figure 2). Advances in antibody engineering and display technologies have enabled rapid identification of high-affinity VHHs from diverse sources, followed by scalable production and molecular refinement. These processes collectively underpin the versatility and adaptability of VHH-based platforms in infectious disease research.

### 4.1. Library Generation and Construction

The initial step in nanobody development involves the generation of diverse VHH repertoires through immunization or synthetic design, followed by library construction for downstream screening [62,63,64].

Immune libraries are generated by immunizing camelids (e.g., camels, llamas, or alpacas) with a target antigen, such as a viral protein or bacterial toxin [65,66,67,68,69]. Following immunization, peripheral blood lymphocytes are isolated, and the VHH-encoding sequences are amplified from B cells. These libraries are enriched for antigen-specific binders and typically yield high-affinity nanobodies due to in vivo affinity maturation [65,66,67,68,69].

In contrast, naïve libraries are constructed from non-immunized animals and represent the natural baseline diversity of VHH sequences [62,70,71]. While they lack prior antigen exposure, sufficiently large naïve libraries can still yield functional binders, particularly when combined with robust selection strategies [62,70,71].

Synthetic libraries are engineered in vitro by introducing controlled diversity into key antigen-binding regions, particularly the complementarity-determining regions (CDRs), with a focus on CDR3 variability. These libraries allow precise control over sequence composition and can be tailored to enhance stability, solubility, or developability. Synthetic approaches also eliminate the need for animal immunization and enable rapid generation of binders against toxic, unstable, or non-immunogenic [64,72].

### 4.2. VHH Selection Technologies

Following library construction, high-affinity VHHs are isolated using in vitro selection platforms that link genotype to phenotype. Phage display is the most widely used technique for VHH selection. In this system, VHH sequences are displayed on the surface of bacteriophages, allowing iterative rounds of binding (biopanning) against immobilized antigens [19,62,73,74,75]. Non-binding phages are washed away, while high-affinity binders are enriched through successive selection cycles. This method is highly robust, scalable, and compatible with a wide range of targets [19,62,73,74,75].

Yeast display represents an alternative platform in which VHHs are expressed on the surface of yeast cells [24,67,76,77,78]. This system enables quantitative screening using flow cytometry, allowing simultaneous assessment of binding affinity and expression levels. Yeast display is particularly advantageous for fine-tuning binding characteristics and conducting affinity maturation through high-throughput sorting [24,67,76,77,78]. In addition, cell-free systems such as ribosome display and mRNA display have been employed for selection, offering the advantage of extremely large library sizes and rapid in vitro screening without transformation limitations [19,79]. These approaches can facilitate the identification of ultra-high-affinity binders and are particularly useful for difficult or toxic targets. Together, these selection technologies enable the rapid identification of VHHs with desired specificity, affinity, and biophysical properties, forming the foundation for downstream engineering.

### 4.3. Recombinant Expression and Production

Once selected, VHHs can be produced recombinantly using a variety of expression systems, each offering distinct advantages depending on the intended application. *. coli* is the most commonly used host for VHHproduction due to its simplicity, rapid growth, and cost-effectiveness [80,81,82]. VHHs can be expressed in the periplasmic space to facilitate proper folding and disulfide bond formation, yielding high amounts of functional protein [80,81,82].

Yeast expression systems (e.g., *Pichia pastoris*) provide an alternative platform that combines relatively high yields with eukaryotic protein processing capabilities [24,36,78,83]. Yeast systems are particularly useful when enhanced secretion or post-translational modifications are desired [24,36,78,83].

Mammalian expression systems are typically employed for more complex constructs, such as Fc-fusion proteins or multivalent formats requiring glycosylation [9,24,84]. Although more resource-intensive, these systems ensure proper folding, stability, and functional activity for clinical-grade production [9,24,84].

The flexibility in expression platforms allows VHH to be produced efficiently at both research and industrial scales, supporting their broad applicability. Plant-based expression systems have emerged as a promising and scalable alternative for nanobody production [26,85]. Platforms such as *Nicotiana benthamiana* enable rapid, transient expression of recombinant VHHs using agroinfiltration techniques [26,85]. Plant systems offer several advantages, including low production costs, scalability, and reduced risk of contamination with human pathogens [26,85]. They are particularly attractive for large-scale manufacturing in response to emerging infectious diseases. Furthermore, plant expression allows for the production of functional VHH constructs, including multivalent and Fc-fused formats, although differences in glycosylation patterns compared to mammalian systems may require additional optimization for clinical applications [86]. The flexibility in expression platforms allows VHHs to be produced efficiently at both research and industrial scales, supporting their broad applicability.

### 4.4. Engineering and Optimization

Following selection and expression, VHHs often undergo further optimization to enhance their functional performance and pharmacokinetic properties. Affinity maturation involves the introduction of targeted mutations within the antigen-binding regions, followed by re-selection to improve binding strength and specificity. This can be achieved through error-prone PCR, site-directed mutagenesis, or display-based evolution strategies. Enhanced affinity is particularly important for therapeutic applications requiring potent neutralization of microbial targets [87,88].

Half-life extension strategies are employed to address the rapid renal clearance associated with the small size of VHHs. One common approach is Fc fusion, where the VHH is genetically linked to the Fc region of an immunoglobulin, thereby increasing molecular size and enabling engagement with neonatal Fc receptors (FcRn) for prolonged circulation. Additionally, Fc fusion can confer effector functions such as antibody-dependent cellular cytotoxicity [34].

Alternative approaches include PEGylation, which involves the covalent attachment of polyethylene glycol chains to increase molecular size and reduce renal filtration, and albumin binding, achieved either through fusion to albumin-binding domains or direct interaction with serum albumin. These strategies significantly extend systemic half-life while maintaining antigen-binding functionality [33,35,36,89].

## 5. VHHs Against Viral Infections

VHHs demonstrate potent antiviral effectiveness against both respiratory and non-respiratory viruses, with neutralization achieved at picomolar to low nanomolar concentrations across diverse viral families. VHH antiviral mechanisms included blocking viral entry by targeting spike proteins [90], hemagglutinin [91], or envelope glycoproteins [92]; inhibiting replication by preventing nuclear import of viral ribonucleoproteins [93] or targeting viral polymerases [94]; and interfering with essential viral proteins like NS3/4A protease [95].

Prophylactic treatment was consistently more effective than therapeutic administration [96,97,98], though some VHHs retained activity when given 16–24 h post-infection [90]. Their small size, structural flexibility, and ability to target conserved or cryptic epitopes enable them to neutralize viruses through diverse mechanisms. In addition, their compatibility with non-invasive delivery routes and advanced engineering strategies has accelerated their development as next-generation antiviral biologics.

**Figure 3:**
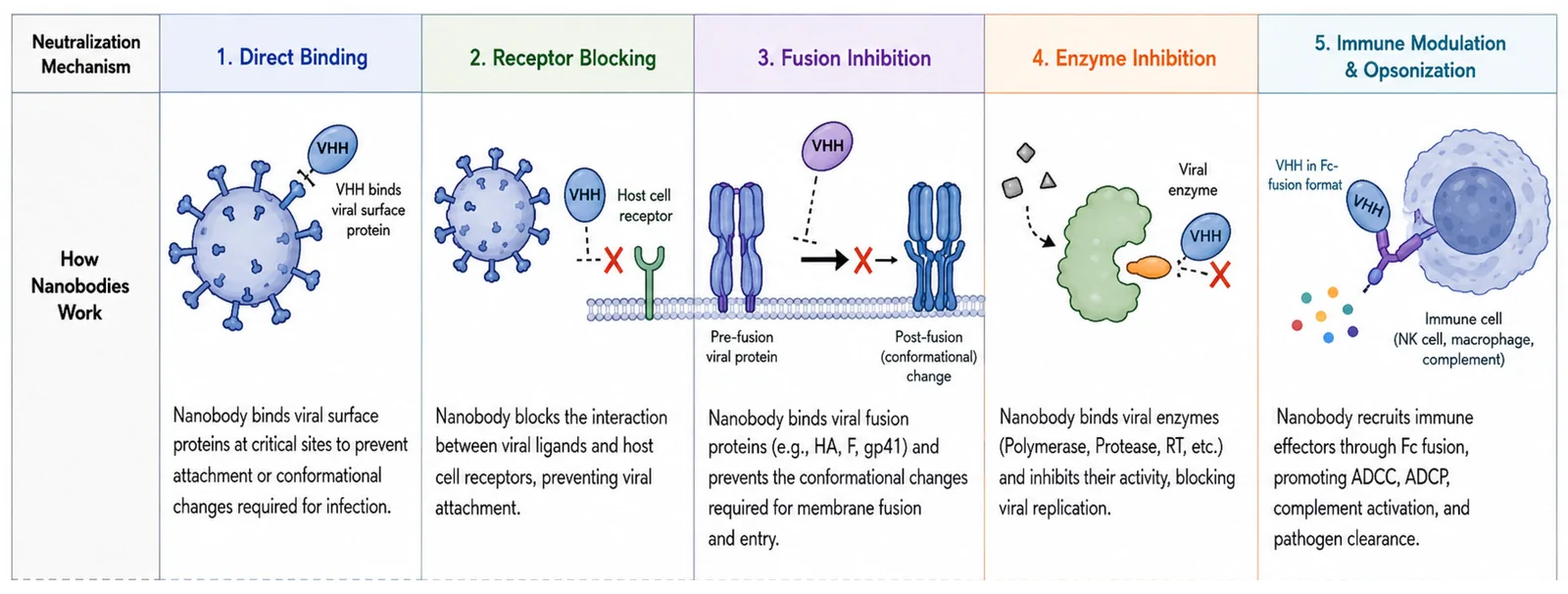
Mechanisms of antiviral action of camelid VHH. (1) Direct binding: VHHs bind critical epitopes on viral surface proteins, preventing attachment or inducing conformational constraints that impair infection. (2) Receptor blocking: VHHs disrupt interactions between viral ligands and host cell receptors, thereby preventing viral attachment and entry. (3) Fusion inhibition: VHHs target viral fusion proteins, such as hemagglutinin (HA) or respiratory syncytial virus fusion protein (F), and prevent the conformational changes required for membrane fusion and viral entry. (4) Enzyme inhibition: VHHs targeting viral enzymes, including polymerases, proteases, or reverse transcriptase, can inhibit enzymatic activity and consequently suppress viral replication. (5) Immune modulation and opsonization: engineered VHH formats containing an Fc region can recruit Fc dependent immune effector mechanisms, including antibody dependent cellular cytotoxicity (ADCC), antibody dependent cellular phagocytosis (ADCP), and complement activation, thereby promoting clearance of virus or infected cells. These mechanisms may operate individually or in combination depending on the viral target, epitope, and VHH format.

The diversity of viral targets, VHH format, expression system, and impact on viral replication across various viral respiratory and non-respiratory pathogens is summarized in Table 1. Furthermore, the main potential mechanisms underlying VHH antiviral activities are illustrated in Figure 3, providing a conceptual framework for understanding their broad-spectrum antiviral potential.

### 5.1. Blocking Viral Entry and Fusion

A major mechanism underlying the antiviral activity of VHHs involves inhibition of viral attachment and membrane fusion. Numerous VHHs targeting coronaviruses, influenza viruses, respiratory syncytial virus (RSV), and HIV-1 function by interfering with receptor engagement or fusion-associated conformational changes [90,92,98].

**Table 1:** Representative Camelid VHH Targeting Viral Pathogens.

Study	Virus Targeted	Target Antigen	VHH Identifier	VHH Format	Expression System	Viral Function Inhibited	Mechanistic Observation in Study	Reported Impact on Viral Replication/Prophylactic Efficacy
Guangyu Zhao et al., 2018 [99]	MERS-CoV	RBD of spike protein	NbMS10, NbMS10-Fc	Fc-tagged	Yeast (extracellular)	Spike RBD engagement with host receptor (entry)	Binds conserved RBD receptor-binding interface and blocks receptor binding.	Complete protection of humanized mice from lethal MERS-CoV challenge with NbMS10-Fc; cross-neutralized diverse isolates.
V. Stalin Raj et al., 2018 [100]	MERS-CoV	RBD of spike protein	VHH-1, VHH-4, VHH-83, VHH-101	Monovalent	*E. coli*	Spike RBD–DPP4 engagement (entry)	RBD-targeting VHHs; neutralizing activity is consistent with receptor-attachment blockade.	Protection against MERS-CoV infection reported in the study; quantitative replication outcome not added here.
F. Idoudi et al., 2026 [101]	MERS-CoV	MERS-CoV RBD	VHH-227, VHH-T71	Fc fusion	Extracellular	Spike RBD–DPP4 engagement (entry)	VHH-227 fully blocks DPP4 binding; VHH-T71 recognizes the RBD core subdomain.	VHH-227 achieved complete neutralization; VHH-T71 achieved partial neutralization (~80%) of RBD mutants in vitro. No animal efficacy reported.
Bert Schepens et al., 2021 [90]	SARS-CoV-1, SARS-CoV-2	RBD of spike protein	VHH72	Monovalent and bivalent	CHO cells *P. pastoris*	Spike-mediated receptor engagement/entry	Recognizes a conserved, sterically restricted RBD epitope; Fc fusion affinity-enhanced.	Controlled SARS-CoV-2 replication in prophylactic and therapeutic mouse and hamster models; also neutralized SARS-CoV-1/2.
Kai Wang et al., 2025 [102]	SARS-CoV-2	S1 protein	VHH21	Dimeric	*E. coli*	Spike conformational transition/fusion	VHH21 drives an irreversible postfusion spike transition and destabilizes fusion; its epitope is distinct from the ACE2-binding site.	Neutralized pseudovirion infection across tested variants. Viral replication and animal prophylaxis were not reported.
Jessica Hong et al., 2022 [103]	SARS-CoV-2	S1 and S2 subunits	8A2, 7A3	Fc fusion	Phage display	Spike-mediated entry/fusion	8A2 binds S1; 7A3 reaches a buried epitope extending into S2.	Broadly neutralized variants and protected mice from lethal B.1.351 or B.1.617.2 challenge; the abstract does not define complete versus partial in-vitro inhibition.
J. Gai et al., 2020 [104]	SARS-CoV-2	SARS-CoV-2 RBD	Nb11-59	Monovalent	*P. pastoris*	RBD–ACE2 engagement (entry)	RBD-directed VHH blocks ACE2 binding.	Potent in-vitro neutralization reported; prophylactic efficacy was not established in this study.
Bert Schepens et al., 2011 [98]	RSV	RSV F protein	F-VHHb	Bivalent	Extracellular	F-protein-mediated membrane fusion	Bivalent F-VHHb inhibited fusion without affecting viral attachment.	Intranasal prophylaxis protected mice; post-infection treatment reduced viral replication and lung inflammation (partial therapeutic suppression).
Li Ma et al., 2023 [105]	RSV	Prefusion RSV F	F-VHH-L66	Monovalent	Expi293 cells	Prefusion F-mediated membrane fusion/entry	F-VHH-L66 is specific for trimeric prefusion RSV F.	Neutralized RSV(A) with efficacy similar to AM14 in the reported bioactivity assay; no animal prophylaxis study reported.
Xiuqin Huang et al., 2024 [91]	Avian influenza H7N9	H7 hemagglutinin	Nb-Z77	Oligomeric forms	Yeast	HA-mediated receptor-binding/hemagglutination	Nb-Z77 binds H7 HA; oligomerization improved hemagglutination-inhibition activity.	Oligomeric Nb-Z77 constructs completely inhibited hemagglutination of inactivated H7-Re1 virus at the tested threshold. Viral replication and animal prophylaxis were not reported.
F. M. Cardoso et al., 2014 [106]	H5N1 influenza	Neuraminidase	N1-VHHm, N1-VHHb, N1-VHH-Fc	Monovalent and bivalent	*E. coli*, plants	Neuraminidase enzymatic activity (virion release/spread)	NA-directed VHHs inhibit neuraminidase function.	Protected mice from H5N1 challenge; antiviral activity was format-dependent, so report as protection rather than total inhibition.
Guowei Wei et al., 2011 [107]	Influenza A (H3N2, H1N1)	M2 ion channel	M2-7A	Monovalent	*E. coli*	M2 proton-channel function (uncoating)	M2-7A blocks proton influx through the M2 ion channel.	Inhibited replication of amantadine-sensitive and -resistant influenza A in vitro and protected mice from lethal challenge.
J. Ashour et al., 2014 [93]	Influenza A (PR8)	Nucleoprotein	αNP-VHH1-6	Monovalent	Intracellular	vRNP nuclear import; downstream viral transcription/replication	Intracellular αNP-VHHs disrupt early infection and prevent vRNP nuclear import.	Replication inhibited in cultured cells; not an in-vivo prophylaxis study.
Mélissa Bessonne et al., 2024 [94]	Influenza A (H3N2, H7N1)	PA-PB1 dimer	VHH16	Single-domain	Intracellular	PA–PB1 trafficking/RNA-polymerase function	VHH16 binds PA–PB1 and impairs its nuclear transport, thereby inhibiting polymerase function.	Blocked multiplication of multiple influenza A subtypes in cells, including when induced late after infection.
F. Schmidt et al., 2016 [108]	Influenza A, VSV	Nucleoproteins	Multiple (19 antiviral VHHs)	HA-tagged	Intracellular	Nucleoprotein-dependent vRNP import or mRNA transcription	Intracellular antiviral VHHs prevented vRNP nuclear import (influenza A) or mRNA transcription (VSV).	Nineteen VHHs protected A549 cells from lethal influenza A or VSV infection; no animal prophylaxis was tested.
Kathrin Koch et al., 2017 [92]	HIV-1 subtype C	CD4 binding site	VHH-A6, VHH-28	Monovalent	*E. coli*	Env CD4-binding-site engagement (entry)	Broadly neutralizing VHHs preferentially target the gp120 CD4-binding site.	Six VHH families neutralized heterologous primary isolates; a two-VHH combination covered 19/21 strains in a standardized in-vitro panel. No in-vivo prophylaxis study.
Surasak Jittavisutthikul et al., 2015 [95]	HCV genotypes 2a, 3a	NS3/4A protease	VHH24, VHH28, VHH41	Monovalent	*E. coli*	NS3/4A protease activity	Transbodies bind residues in the protease catalytic triad/oxyanion loop and NS4A-cofactor interface.	Reduced intracellular and extracellular HCV RNA, HCV foci, and supernatant core antigen in Huh7 cells; no complete-inhibition claim or animal prophylaxis study.
S. Terryn et al., 2014 [97]	Rabies virus	Rabies glycoprotein G	Rab-E8/H7	Biparatopic Multivalent	*E. coli,* *P. pastoris* Ablynx production	Glycoprotein-G-mediated entry	Rab-E8/H7 is a biparatopic G-directed neutralizing VHH construct.	Post-exposure treatment prolonged survival; albumin-binding construct completely rescued 43% of mice, not universal protection.

In coronaviruses, VHHs targeting the receptor-binding domain (RBD) effectively prevent host receptor interaction. SARS-CoV-2-specific VHH72 binds a highly conserved RBD epitope, thereby blocking ACE2 engagement and stabilizing the spike protein in a nonproductive conformation [90]. Similarly, MERS-CoV-targeting VHHs inhibit viral attachment through blockade of DPP4 binding, with several constructs demonstrating potent neutralization at picomolar concentrations and complete protection in animal models [99,101]. Importantly, some VHHs retain activity against multiple SARS-related coronaviruses and emerging variants, reflecting recognition of structurally conserved epitopes [102].

A comparable mechanism is observed in RSV, where F-protein-specific nanobodies inhibit viral fusion following attachment [98]. Bivalent RSV VHH constructs demonstrated substantially greater potency than conventional monoclonal antibodies, significantly reducing viral replication and providing effective prophylactic protection following intranasal administration [98]. Notably, several VHHs exhibit specificity for the prefusion conformation of RSV F, a highly conserved and neutralization-sensitive structural state [105].

Influenza-targeting VHHs similarly interfere with viral entry processes. VHHs directed against hemagglutinin inhibit hemagglutination and prevent viral attachment [91], while those targeting the M2 ion channel block proton influx required for viral uncoating [107]. In HIV-1, VHHs recognizing the CD4-binding site prevent Env–CD4 interactions, thereby suppressing viral entry into host cells [92].

### 5.2. Intracellular Neutralization of Viral Replication

**Figure 4:**
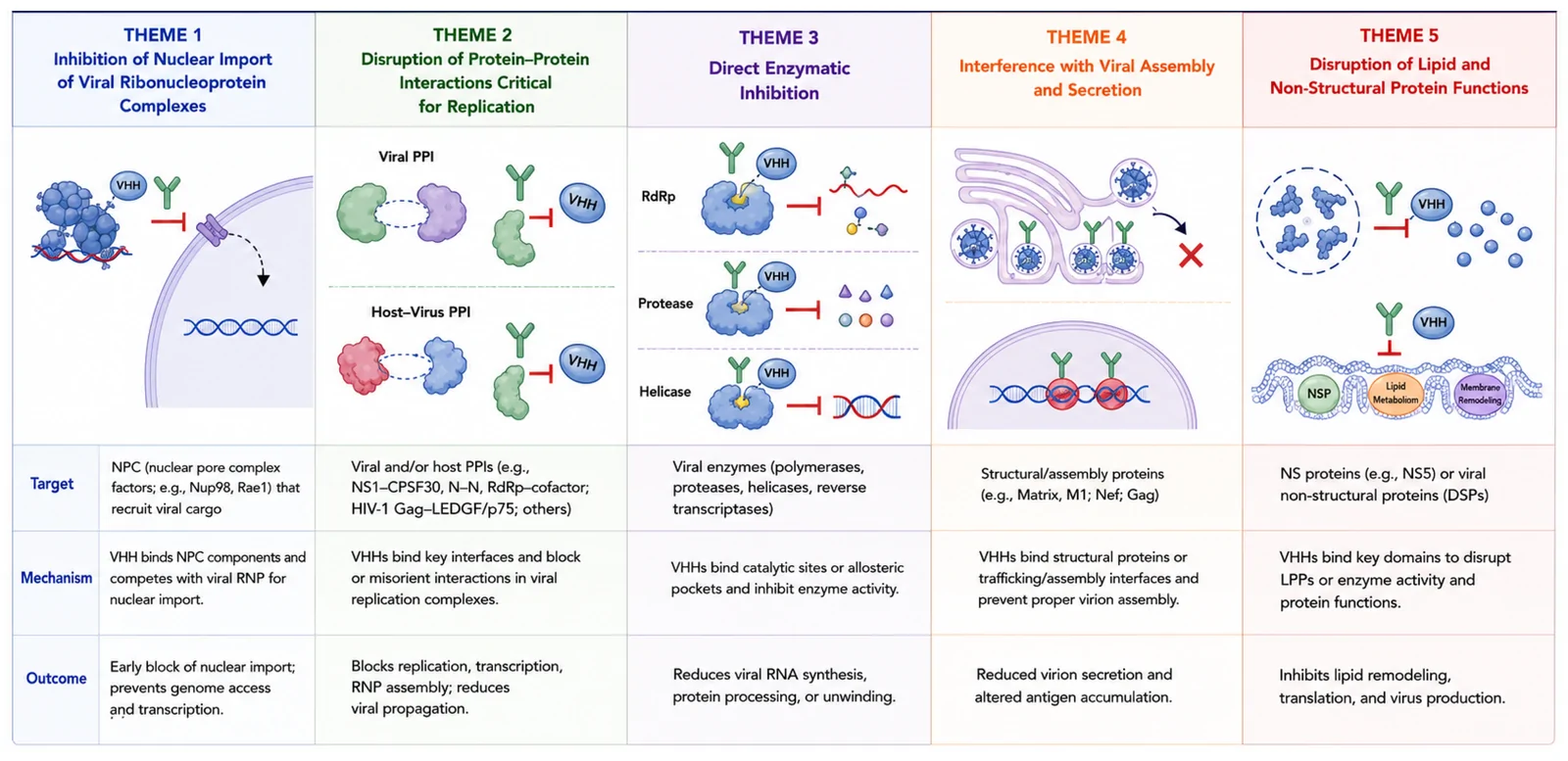
Main intracellular neutralization mechanisms of viruses by camelid VHH. VHH can target intracellular viral or host factors involved in multiple stages of the viral replication cycle. (1) Inhibition of nuclear import: VHHs targeting components of viral ribonucleoprotein complexes or nuclear pore complex associated factors can interfere with nuclear trafficking, thereby restricting genome access and transcription. (2) Disruption of protein–protein interactions: VHHs can bind critical interfaces within viral protein complexes or host virus protein interactions required for replication, transcription, and ribonucleoprotein assembly. (3) Direct enzymatic inhibition: intracellular VHHs can target viral enzymes, including RNA dependent RNA polymerases, proteases, helicases, and reverse transcriptases, and inhibit their catalytic or regulatory functions. (4) Interference with viral assembly and secretion: VHHs directed against structural or assembly proteins can disrupt intracellular trafficking and assembly processes, reducing formation and release of progeny virions. (5) Disruption of lipid and nonstructural protein functions: VHHs can target viral nonstructural proteins or proteins involved in lipid associated replication processes, potentially interfering with membrane remodeling, viral protein function, translation, and virus production.

Beyond extracellular neutralization, VHHs can also interfere with several intracellular stages of viral replication (Figure 4). A prominent mechanism is the inhibition of nuclear trafficking of viral replication complexes. In influenza A virus, intracellular VHHs targeting the nucleoprotein (NP) prevent nuclear import of incoming viral ribonucleoprotein (vRNP) complexes, thereby blocking an early post-entry step in viral replication [93,108,109]. Interestingly, these VHHs inhibit nuclear import of NP only when incorporated within the vRNP complex, while leaving the transport of NP expressed alone unaffected, suggesting selective recognition of the assembled replication complex and distinct nuclear localization mechanisms during different stages of infection [93]. Similar effects have been demonstrated against the influenza polymerase, where VHH16 targets the PA–PB1 dimer interface and disrupts nuclear transport of the polymerase complex while simultaneously interfering with interactions required for transcription and genome replication [94]. Its exceptionally high affinity and slow dissociation kinetics contribute to sustained antiviral activity even when expression is initiated several hours after infection [94]. Likewise, the influenza NP-specific αNP-VHH1 binds an epitope overlapping determinants recognized by cellular Mx GTPases, providing structural evidence that VHH-mediated inhibition can mimic innate antiviral defense mechanisms [109].

Intracellular VHHs also inhibit viral replication by disrupting protein–protein interactions essential for viral replication, transcription, and genome integration. In HIV-1, the Nb190 binds the N-terminal multimerization domain of the Rev protein, preventing Rev oligomerization on the Rev response element and thereby blocking CRM1-mediated nuclear export of viral RNA [110,111]. Stable intracellular expression of Nb190 markedly suppresses replication across multiple HIV-1 subtypes, with only rare naturally occurring escape variants identified [79]. Similar disruption of viral protein interactions has been described in vesicular stomatitis virus (VSV), where VHH1307 competitively blocks binding of the phosphoprotein cofactor (P) to the nucleoprotein (N), inhibiting both genome replication and viral transcription, whereas VHH1004 selectively impairs genome replication by targeting a distinct region of N [112].

Direct inhibition of viral enzymes represents another major antiviral mechanism. Several HCV-targeting VHHs bind functional regions of NS5B RNA-dependent RNA polymerase, preventing template and substrate access to the catalytic cleft [95,113]. Additional VHHs targeting the NS3/4A protease interact directly with catalytic residues and the oxyanion loop, suppressing viral RNA synthesis and viral protein production [95]. Likewise, VHHs directed against the NS3 helicase inhibit essential domains involved in ATP-dependent helicase activity, enzyme folding, and nucleic acid binding [114].

Several intracellular VHHs interfere with later stages of the viral life cycle by preventing viral assembly, maturation, and secretion. In hepatitis B virus (HBV), endoplasmic reticulum-retained intrabodies targeting HBsAg sequester viral envelope proteins within the secretory pathway, markedly reducing virion release in vitro and in vivo [115]. Additional HBcAg-specific intrabodies alter intracellular localization of viral proteins, although the underlying mechanism remains incompletely understood [115].

Recent studies have also identified novel antiviral mechanisms involving disruption of viral biomolecular condensates and non-structural protein function. VHHs directed against multiple domains of the SARS-CoV-2 nucleocapsid protein inhibit liquid–liquid phase separation (LLPS), thereby disrupting condensate formation required for efficient viral genome organization and replication [116]. Comparable intracellular antiviral activity has been demonstrated against other emerging viruses. VHHs targeting the nucleocapsid protein of porcine deltacoronavirus (PDCoV) reduce viral RNA synthesis and infectious virus production [117], whereas VHHs directed against porcine reproductive and respiratory syndrome virus (PRRSV) nonstructural proteins Nsp9 and Nsp4 effectively suppress genome replication and transcription [118,119]. Similarly, an intracellular VHH targeting the NS4B protein of bovine viral diarrhea virus (BVDV) almost completely inhibited viral replication, while a cell-penetrating formulation retained substantial antiviral activity following extracellular administration [120].

Beyond direct antiviral activity, several intracellular VHHs contribute to restoration of host antiviral immunity. HCV-targeting VHHs directed against NS3/4A, NS4B, and NS5A not only suppress viral replication but also restore innate immune signaling by upregulating interferon-associated pathways, including TRIF, TRAF3, IRF3, IL-28B, and IFN-β [95,121]. These findings suggest that inhibition of viral immune antagonists allows recovery of host antiviral responses, providing an additional mechanism contributing to the overall antiviral efficacy of intracellular VHH therapy.

### 5.3. Broad Neutralization and Viral Escape

One of the most significant advantages of VHHs is their capacity to recognize conserved epitopes across genetically diverse viral strains. This broad neutralization potential is particularly valuable for highly mutable RNA viruses. Several coronavirus-targeting VHHs exhibit cross-neutralization against SARS-CoV-1, SARS-CoV-2, Bat RaTG13, and multiple emerging variants by targeting conserved RBD regions [90,102]. Similarly, MERS-CoV VHHs demonstrate broad activity against strains originating from both humans and camels, including RBD mutants associated with immune escape [100,101].

In influenza viruses, VHHs targeting conserved neuraminidase or M2 epitopes retain activity against antiviral-resistant strains, including oseltamivir-resistant H5N1 viruses and amantadine-resistant variants [106,107]. Poliovirus-targeting VHHs also inhibit viral replication through binding of structurally critical capsid epitopes [122,123]. Notably, studies reported an inability to generate escape mutants against specific VHHs, suggesting that they target highly constrained regions essential for viral fitness [122,123]. In rabies virus, biparatopic constructs recognizing multiple glycoprotein epitopes significantly reduced the likelihood of escape, while poliovirus studies demonstrated complete resistance to escape mutant generation against certain VHHs [69,97,122,123]. These findings collectively highlight the ability of VHHs to target structurally constrained regions that are less permissive to mutation, thereby reducing viral escape potential and enhancing long-term therapeutic effectiveness.

However, VHHs, like conventional neutralizing antibodies, impose selective pressure on viral populations and can select escape variants. Escape may arise through amino-acid substitutions within the epitope that directly disrupt paratope contacts; substitutions at adjacent residues that alter local conformation or glycan shielding; and more distal changes that shift the accessibility or conformational equilibrium of the targeted viral protein. Escape is especially plausible when a monovalent VHH relies on a single exposed, variable epitope [124,125,126].

The risk is clinically relevant during prolonged viral replication under selective pressure. Treatment-emergent anti-spike monoclonal-antibody resistance was reported most often in immunocompromised patients with high and persistent SARS-CoV-2 replication who received antibody monotherapy [127]. This experience argues against describing VHHs as inherently escape resistant. Rather, VHHs directed to conserved, functionally constrained epitopes may have a higher barrier to resistance. That advantage must be demonstrated experimentally for each VHH–virus pair through deep mutational scanning or serial-passage selection, followed by confirmation of both loss of susceptibility and the escape variant’s replicative fitness.

Several design strategies can reduce, but not eliminate, escape risk. First, structural and functional mapping should prioritize conserved epitopes whose alteration carries a measurable fitness cost. Second, two or more VHHs recognizing non-overlapping epitopes can be co-administered or fused as biparatopic/multispecific constructs; viral escape then requires compatible changes at more than one site. In a SARS-CoV-2 evolution experiment, a single multivalent VHH format still selected fully resistant single-amino-acid escape mutants, whereas a biparatopic construct directed to two independent epitopes largely prevented escape [128]. Third, combining an entry-directed VHH with a mechanistically orthogonal low-molecular-weight direct-acting antivirals (e.g., replication-enzyme inhibitor) may further increase the resistance barrier. In fact, low-molecular-weight direct-acting antivirals remain indispensable for many viral diseases and they generally lower manufacturing costs and straightforward large-scale production [129,130] VHHs may therefore be viewed as complementary rather than intrinsically superior to small molecules.

### 5.4. Unconventional and Emerging Antiviral Mechanisms

Beyond classical neutralization, VHHs can exert antiviral activity through unconventional mechanisms [102]. A notable example is VHH21, which functions as a “abzyme-like nanobody” capable of catalyzing irreversible postfusion conformational changes within the SARS-CoV-2 spike protein, resulting in destruction of the spike trimer rather than simple receptor blockade [102]. Such findings underscore the mechanistic diversity of VHH-mediated antiviral activity and highlight their potential to exploit noncanonical pathways inaccessible to conventional antibodies.

Taken together, camelid VHHs represent a highly versatile antiviral platform capable of targeting multiple stages of viral infection through diverse and mechanistically sophisticated pathways. Their broad neutralization capacity, higher resistance to viral escape, engineering flexibility, and compatibility with alternative delivery strategies position them as promising candidates for next-generation antiviral therapeutics and pandemic preparedness platforms.

## 6. Clinical Translation of Antiviral VHH Candidates

Clinical translation of antiviral VHHs remains limited (Table 2). ALX-0171, an inhaled trivalent VHH targeting respiratory syncytial virus (RSV) F protein, is the most developed antiviral example identified in the current literature and trial registry. Its development program included completed Phase I safety and pharmacokinetic studies, a completed Phase 1/2 study in hospitalized infants and toddlers, and a completed international Phase 2b dose-ranging trial; a Phase 2 study in hematopoietic stem-cell transplant recipients was withdrawn, and a Japanese Phase 2 study was terminated early [131,132,133,134,135,136,137].

In the Phase 2b randomized trial, nebulized ALX-0171 accelerated reduction of infectious RSV by plaque assay but did not improve prespecified clinical outcomes relative to placebo in hospitalized children. This discordance highlights a central translational lesson: antiviral activity measured after established lower-respiratory-tract disease may not translate into clinical benefit when inflammatory pathology is already prominent [135,138].

**Table 2:** Clinical development of VHH-based therapeutics against viral infections..

Program/Virus	Registry ID; Phase; *n*	Population	Route and Regimen	Status/Timing	Key Assessment	Ref.
ALX-0171 RSV	NCT01483911 Phase 1; *n* = 60	Healthy adult men, 18–55 years	Pulmonary inhalation; single/multiple ascending dose	Completed Nov 2011–May 2012	Safety/tolerability and plasma pharmacokinetics; no registry results posted.	[131]
ALX-0171 RSV	NCT01875926 Phase 1; *n* = 44	Healthy adult men, 18–55 years	Inhaled and IV dosing	Completed May–Oct 2013	Pulmonary/systemic PK, local tolerability and anti-drug antibodies; no registry results posted.	[133]
ALX-0171 RSV	NCT01909843 Phase 1; *n* = 24	Adults with hyperresponsive airways, 18–60 years	Escalating inhaled dosing	Completed Jul–Dec 2013	Bronchoconstriction, safety, PK and immunogenicity; no registry results posted.	[132]
ALX-0171 RSV	NCT02309320 Phase 1/2; *n* = 53	Hospitalized, otherwise healthy infants/toddlers, 28 days–23 months	Inhaled ALX-0171 vs. placebo; dose not reported in registry summary	Completed Dec 2014–Feb 2016	Safety/tolerability primary; clinical activity, viral-load PD, PK and immunogenicity secondary. No registry results posted.	[134]
ALX-0171 RSV	NCT02979431 Phase 2b; *n* = 180	Hospitalized infants/toddlers, 28 days–<2 years; 16 countries	Nebulized 3, 6 or 9 mg/kg once daily for 3 days + standard care; placebo-controlled	Completed Jan 2017–May 2018	Infectious RSV clearance was faster, but clinical response time and global severity did not improve versus placebo.	[135]
ALX-0171 RSV	NCT03418571 Phase 2; *n* = 15	Hospitalized Japanese infants/young children, 28 days–<2 years	Inhaled 1.5 mg/kg once daily for 3 days; placebo-controlled	Terminated Mar–Oct 2018	Only the first cohort completed; higher planned doses were not studied. Sponsor record links termination to discontinuation following the Phase 2b program.	[136]
ALX-0171 RSV after HSCT	NCT03468829 Phase 2; *n* = 0	Adults (18–75 years) with RSV after hematopoietic stem-cell transplantation	Two inhaled ALX-0171 dose arms or placebo, daily up to 14 days	Withdrawn Scheduled 2019–2020	Planned viral-load, clinical and safety endpoints; no enrolled participants, so no treatment inference.	[137]
VHH batch 203027 Rotavirus	NCT01259765 Phase 2; *n* = 176	Hospitalized male children, 6–24 months; Bangladesh	Oral rehydration solution: 165 mg VHH + maltodextrin; placebo-controlled	Completed Jan 2006–Nov 2009	Stool volume/severity, diarrhoea duration, and faecal viral excretion. Registry links the subsequent randomized report of reduced stool output.	[139]

In enteric infection, VHH batch 203027 demonstrated a complementary oral-format strategy: a randomized Phase 2 study tested a llama/camel-derived VHH fragment in oral rehydration solution in 176 children with rotavirus diarrhoea, with stool output, diarrhoea duration, and viral shedding as primary efficacy measures; the registry cites a subsequent randomized publication reporting reduced stool output [139,140]. Together, these cases show that VHH stability and local delivery can support clinical dosing by inhalation or orally, while also showing the central translational challenge: robust target-proximal antiviral activity must still be linked to a patient-relevant benefit, likely through earlier treatment, the right infection compartment, and appropriately sensitive clinical endpoints.

## 7. Future Perspectives and Emerging Directions

VHHs are emerging as a highly versatile platform for combating infectious diseases due to their small size, high specificity, remarkable stability, and ability to bind cryptic or conserved epitopes inaccessible to conventional antibodies. Their applications extend across various viral infections with different mechanisms of action.

Despite these promising attributes, several challenges remain before VHHs can achieve widespread clinical translation. Their small molecular size results in rapid renal clearance and short plasma half-life, often necessitating engineering approaches such as Fc fusion or albumin-binding domains to improve pharmacokinetic performance [32,33,34,35,36]. In addition, VHHs naturally lack the Fc region responsible for immune effector functions, limiting their ability to recruit mechanisms such as antibody-dependent cellular cytotoxicity unless Fc-containing formats are employed [141,142]. Another important consideration is that monovalent VHHs may exhibit limited neutralization potency or breadth against genetically diverse viral strains and emerging variants, highlighting the importance of multivalent, bispecific, and other engineered constructs [141,143]. Encouragingly, accumulating evidence indicates that these limitations can be effectively mitigated through rational molecular engineering, allowing VHHs to retain their unique advantages while improving their therapeutic efficacy and clinical applicability.

The integration of VHHs with nanotechnology enables targeted delivery systems using nanoparticles, liposomes, and biosensors [52]. These strategies improve therapeutic precision, enhance drug bioavailability, reduce off-target effects, and support advanced diagnostic applications [52].

Artificial intelligence (AI) and machine learning are also transforming VHH development by accelerating epitope mapping, affinity maturation, and structural optimization [144]. AI-guided computational approaches can rapidly identify conserved pathogen targets, facilitating the development of broadly neutralizing nanobodies and significantly shortening discovery timelines during emerging outbreaks [144].

In addition, the adaptability of camelid VHHs strongly aligns with the One Health framework, supporting applications in human medicine, veterinary medicine, and environmental surveillance [6,145,146]. Their stability and scalability make them suitable for deployment in diverse ecological settings, including zoonotic disease monitoring and pathogen detection in water, food, and air systems.

Importantly, camelid VHHs represent promising tools for pandemic preparedness [52,147]. Rapid screening of immune or synthetic VHH libraries against emerging pathogens enables fast identification of high-affinity binders [8,63]. Combined with scalable microbial production, aerosolized delivery systems, and AI-assisted design, VHHs could form part of next-generation rapid-response platforms against future pandemics [7,12].

Overall, camelid VHHs constitute a promising and adaptable technology with substantial potential to revolutionize infectious disease diagnostics, therapeutics, surveillance, and global public health strategies. The current evidence, however, supports cautious interpretation of antiviral VHH clinical readiness and prioritizes earlier intervention, clinically meaningful endpoints, and exposure–response analyses in future studies.

## Data Availability

No new data were created or analyzed in this study. Data sharing is not applicable to this article.
